# HIF-1α Promotes the Metastasis of Esophageal Squamous Cell Carcinoma by Targeting SP1

**DOI:** 10.7150/jca.35537

**Published:** 2020-01-01

**Authors:** Xueting Hu, Jiatong Lin, Ming Jiang, Xiaotian He, Kefeng Wang, Wenjian Wang, Chuwen Hu, Zhiwen Shen, Zhanghai He, Huayue Lin, Duoguang Wu, Minghui Wang

**Affiliations:** 1 Guangdong Provincial Key Laboratory of Malignant Tumor Epigenetics and Gene Regulation, Sun Yat-Sen Memorial Hospital, Sun Yat-Sen University, Guangzhou, China 510120.; 2 Department of Thoracic Surgery, Sun Yat-Sen Memorial Hospital, Sun Yat-Sen University, Guangzhou, China 510120.; 3 Department of Thoracic Surgery, Affiliated Cancer Hospital & Institute of Guangzhou Medical University, Guangzhou, China 510120.; 4 Breast Tumor Center, Sun Yat-Sen Memorial Hospital, Sun Yat-Sen University, Guangzhou, China 510120.

**Keywords:** HIF-1α, SP1, ESCC, tumor metastasis

## Abstract

**Background:** In microenvironment of malignant tumors, Hypoxia-Inducible Factors (HIF), most importantly HIF-1α, play an important role in regulation of adaptive biological response to hypoxia, promoting angiogenesis and metastasis. However, the underlying mechanism that HIF-1α regulates metastasis needs to be further clarified.

**Methods:** The expressions of HIF-1α and SP1 were detected in 182 samples of esophageal squamous cell carcinoma (ESCC) and adjacent normal tissues by immunohistochemistry (IHC), and the correlation between the expression levels of HIF-1α and SP1 was analyzed. The expression of HIF-1α in ESCC cell lines TE1 and KYSE30 was then detected using qRT-PCR and western blot. The potential binding sites of HIF-1α on the *SP1* promoter were analyzed using UCSC and JASPAR databases, verified by chromosomal immunoprecipitation (ChIP) assay and qRT-PCR. The effects of HIF-1α and SP1 on ESCC cell migration and invasion were then tested with Transwell and Matrigel experiments.

**Results:** The expression of HIF-1α in cancer tissues is higher than adjacent normal tissues, and is correlated with metastasis, recurrence and poor prognosis. Upon silencing HIF-1α by siRNA, the invasion and migration ability of ESCC cells were significantly inhibited, which could be restored by the overexpression of SP1. Hypoxic conditions significantly increased the expression of HIF-1α and SP1 at both protein and mRNA levels in ESCC cells. HIF-1α enhanced *SP1* transcription through binding to the promoter region. The expression of protein and mRNA levels of SP1 was decreased by silencing HIF-1α in cells. In contrast, overexpression of HIF-1α significantly increased the mRNA and protein levels of SP1. The expression of SP1 in ESCC was positively correlated with the protein expression of HIF-1α and poor prognosis.

**Conclusion:** The results of our study indicate that HIF-1α promotes metastasis of ESCC by targeting SP1 in a hypoxic microenvironment. Further study on this mechanism may elucidate the possibility of HIF-1α and SP1 as new targets for the treatment of ESCC.

## Introduction

Esophageal squamous cell carcinoma (ESCC) is one of the most common malignant tumors of digestive tract in the world 1. Based on histopathology, esophageal cancer is usually classified into squamous cell carcinoma and adenocarcinoma, with squamous cell carcinoma accounting for about 90% of patients in China 2. Despite the continuous improvement in the diagnosis and treatment, the prognosis of patients with esophageal cancer remains poor, with the overall 5-year survival rate of 15%-34% 3, 4. At present, surgery remains to be the major treatment of esophageal cancer. Combination with preoperative neoadjuvant or postoperative adjuvant radiotherapy and chemotherapy has improved patient prognosis at certain level, however the overall effects of treatment are still unsatisfactory. One of the major factors for poor prognosis of esophageal cancer patients is the tumor metastasis 5-7. Searching of novel biomolecules related to tumor metastasis and clarification of underlying molecular mechanisms could facilitate the screening of patients with high risk of recurrence, as well as the identification of new therapeutic target, therefore would further improve the clinical treatment of esophageal cancer.

Similar to most solid tumors, insufficient oxygen supply often results in a hypoxic microenvironment in esophageal cancer tissue 8. Hypoxia-Inducible Factors (HIF) are important regulators of adaptive response to hypoxia. As one of the most important regulators, elevated expression of HIF-1α has been identified in esophageal cancer 7, 9, 10, prostate cancer 11, lung cancer 12, breast cancer 13 and other malignant tumors. It has been reported that HIF-1α could stimulate tumor angiogenesis and lymphangiogenesis, and promote metastasis and recurrence 14-17. However, the exact functional mechanism of HIF-1α in metastasis of ESCC still needs to be further explored.

It has been reported that specific protein 1 (SP1) regulates the expression of various genes, including housekeeping genes as well as genes involved in regulation of cell proliferation, apoptosis, embryonic development and other physiological activities 18. The expression and function of SP1 varies in different tumor cells. Multiple studies have confirmed the important regulatory role of SP1 in esophageal cancer 19, pancreatic cancer 20 and breast cancer 21. HIF-1α functions as a transcription factor to regulate the expression of multiple genes 22-24. Database prediction has shown that *SP1* might be a potential target of HIF-1α's regulation, suggesting the function of HIF-1α in promoting tumor development and metastasis may through the regulation of SP1.

Studies of HIF-1α and SP1 in tumor metastasis are rare and related mechanisms remain unclear. This study showed that the HIF-1α protein level was higher in cancer tissues than in adjacent normal tissues, and the expression of HIF-1α was correlated with tumor metastasis, recurrence and poor prognosis in patients with esophageal cancer. In addition, HIF-1α, bound to the *SP1* promoter, regulated *SP1* transcription, thereby inducing changes in migration and invasion abilities of esophageal cancer cells. There was a positive correlation between SP1 and HIF-1α protein expression in ESCC samples, and SP1 expression was also correlated with tumor metastasis, recurrence and poor prognosis. In conclusion, the study provided evidence for the molecular mechanism that HIF-1α promotes the metastasis of ESCC through targeting *SP1* transcription. The results indicate the possibility for HIF-1α and SP1 as prognostic factors of ESCC.

## Materials and Methods

### Clinical samples and data collection

Cancer tissue specimens and paraffin sections of adjacent tissues were collected from 182 patients with ESCC who were treated with thoracic surgery at Sun Yat-Sen Memorial Hospital of Sun Yat-Sen University between January 2010 and January 2013. Diagnosis of ESCC for all patients were pathologically confirmed. No patient underwent chemotherapy or radiotherapy before surgery. The usage of patients' tissues was approved by the Ethics Committee of Sun Yat-Sen Memorial Hospital of Sun Yat-Sen University, and informed consents were acquired from all the patients. The clinical pathology and other clinical features of these patients were collected from electronic medical records.

### Immunohistochemistry

Surgically removed cancer and metastatic lymph node tissues were immediately fixed in 10% formaldehyde, embedded in paraffin, and then sectioned. After dehydration with xylene and series of ethanol, samples were incubated in 3% H_2_O_2_ for 10 min at room temperature, washed with PBS, incubated in antigen retrieval solution (sodium sulphate buffer pH 6.0) for high pressure retrieval, then naturally cooled to room temperature and washed with PBS. After blocking samples with 3% bovine serum albumin for 15 min, SPl antibody (rabbit anti-human, Abcam, USA, 1:100 dilution) was added and samples were incubated at 4 °C overnight. After rinsing samples with PBS, universal immunohistochemical secondary antibody (ZhongshanJinqiao, PV-6000, China) was added and samples were incubated for 30 min at 37 °C. After washing samples with PBS, substrate diaminobenzidine (DAB) was added and staining was controlled with regular microscopy. The samples were then counterstained with hematoxylin. After washing by water and decoloring by l% hydrochloric acid ethanol, the samples were put into tap water for bluing. After dehydrating and transparentizing by series of ethanol and xylene, the specimens were sealed by neutral resins for observation.

The scoring of immunohistochemical staining of SP1 was determined using a double-blinded method. Each slice was observed and scored by two senior pathologists separately using a semi-quantitative scoring method. The effect that brown-yellow particles were observed in the nucleus or the cytoplasm was considered as SP1 positive. The samples were then scored according to the percentage of positive cells: 0 as lower than 10%; 1 as 10% ~ 25%; 2 as 25% ~ 50%; 3 as 50% ~ 75%; and 4 as 75% and above. The staining intensity of positive cells was categorized into 4 levels: no expression (0), weak (1), medium (2), and strong (3 points). The final score was the product of the percentage score and the stain intensity score. A final score of 0-3 was then assigned as low expression, and 4-12 was as assigned high expression (4-6: +, 7-9: ++, 10-12: +++).

### Cell line and culture

The human ESCC cell line TE1 and KYSE30 was acquired from the Cell Bank of the Chinese Academy of Sciences (Shanghai, China). Cells were cultured in Roswell Park Memorial Institute (RPMI) 1640 supplemented with 10% fetal bovine serum (FBS) (Gibco, CA, USA). Cell culture plates were kept in a humidified incubator at 37 ° C with 5% CO_2_. For the hypoxic treatment, cells were cultured in an oxygen-controlled incubator (Heal Force, Shanghai, China) under hypoxia conditions (1% O_2_, 5% CO_2_ and 94% N_2_) for 24 to 48 hours.

### Total RNA extraction and RT-PCR

Total RNA was extracted from ESSC cells using TRIzol Reagent (Invitrogen, Carlsbad, CA, USA). The cDNA was then reverse transcribed using a PrimeScriptTM RT kit with gDNA Eraser (TakaRa, Dalian, China). RT-PCR was performed using SYBR® PremixExTaqTMII (TakaRa, Dalian, China) on a LightCycler® 96SW 1.1 RT-PCR system (Roche, Basel, Switzerland) following the manufacturer's instructions. β-Actin was used as an internal control.

Primers for PCR are: HIF-1a, 5'- TGG CTG CAT CTC GAG ACT TT -3'(forward) and 5'- GAA GAC ATC GCG GGG AC -3'(reverse); SP1, 5'- ACC AAG CTG AGC TCC ATG AT -3'(forward) and 5'- CCT CAG TGC ATT GGG TAC TTC -3' (reverse); β-Actin, 5'-TCG TGC GTG ACA TTA AGG AG-3'(forward) and 5 '-GTC AGG CAG CTC GTA GCT CT-3' (reverse).

### Western blot

Cells were lysed in RIPA buffer (Cell Signaling Technology) containing protease inhibitors. The lysate was centrifuged and the supernatant was collected. Protein concentration was determined using a BCA Protein Assay Kit (Pierce). Aliquots (30-40μg) of protein were separated on 10% SDS-PAGE electrophoresis gel and transferred to PVDF membrane (Immobilon-membrane, Millipore). After washed with Tris buffered saline Tween-20 buffer (TBST; pH 7.5; 10 mM Tris, 150 mM NaCl and 0.1% Tween 20) for 10 min x 3 times, PVDF membrane was blocked in 5% skim milk for 1 hour, then incubated overnight at 4 °C with primary antibodies (anti-β-ACTIN, anti-HIF-1α or anti-SP1; 1:1000, CST, USA). After washed three times with TBST (pH 7.5) at room temperature, the PVDF membrane was incubated for 1 hour at room temperature with the secondary antibody, then washed three times with TBST (pH 7.5) at room temperature for 10 min x 3 times. Proteins were then detected using a chemiluminescence and imaging system (Bio-Rad, USA).

### Transient transfection

*HIF-1α* siRNA1,2 and negative control siRNA were synthesized by GenePharma (Shanghai, China). *SP1* siRNA1,2 and negative control siRNA were synthesized by Ribobio (Ribobio Co., China). Cells were transfected with Lipofectamine 3000 (Invitrogen) according to the manufacturer's instructions. After 24 hours of hypoxia treatment, cells were transfected with HIF-1α siRNA for 12 hours. After 48 hours of transfection, cells were harvested and subjected to Western blot analysis.

The pc-HIF-1α plasmid was synthesized by Sino biological, and the pc-SP1 and pcDNA3.1 plasmids were constructed and synthesized by OBIO Technology (Shanghai). The pcDNA3.1 was used as an empty vector. Transfection was performed using Lipofectamine 3000 and P3000 (Invitrogen) according to the manufacturer's instructions. Cells were treated with plasmid for 12 hours and after 48 hours of transfection, cells were harvested and subjected to Western blot analysis.

### ChIP-PCR assays

Formaldehyde (1% concentration) was added into cultured cells, and the cultured cells was then incubated at 37°C for 10 min. The cells were then washed twice with PBS, collected and treated with TNT lysate solution (20 mmol/L Tris-HCl, pH=7.4, 200 mmol/L NaCl, 1% Triton X-100, 1 mmol/L PMSF and 1% aprotinin) in ice bath. The DNA was sheared into 200-500 bp fragments with ultrasonication and co-immunoprecipitated with 2 μg TS2 antibody. Antibody/protein complexes were collected using Protein A magnetic beads and washed 3 times with ChIP buffer (5% SDS, 1 mmol/L EDTA, 0.5% bovine serum albumin and 40 mmol/L NaHPO4, pH = 7.2). After 1% SDS and 1mmol /L NaHCO3 were used to wash the immunoprecipitant, 200 mmol/L NaCl and RNaseA were added to the immunoprecipitant, and the immunoprecipitant was incubated at 65℃ for 4 h to de-crosslink. The samples were then treated with proteinase K for 2 h, and DNAs were purified using chormatography. The SP1 promoter DNA was detected using PCR amplification. Primers used to amplify the -401 to -142 region of Sp1 promoter are: Forward: 5'-CAGCAAGTCACTCCC-3'; Reverse: 5'-GGAGAGGTGCGCGGC-3'. RT-PCR was performed for samples in 3 groups: IgG group (negative control), Input group (positive control), and IP group.

### Cell migration and invasion detection

Cell migration and invasion were assayed in triplicate using 24-well transwell plates and polycarbonate nucleus filters (Merck Millipore Bioscience, Germany) with 8-μm pore size. The cells were deprived of FBS for 24 hours before each experiment. For the invasion assay, inserts were pre-coated with Matrigel (BD Biosciences, Belgium) diluted 1:10 in serum-free Ham's F10 and Matrigel was allowed to polymerize at 37 °C for 1 hour. The inserts were not coated in the migration assay. Control and treated cells (1 x 10^5^ in 200 μl serum free medium) were seeded in the upper chamber of the insert. The lower chamber was filled with 600 μl of medium containing 20% ​​FBS as an attractant. The invasion assay was allowed to proceed for 24 hours and migration assay was incubated for 12 hours. Any cells remaining on top of the insert are scraped off by scraping. The migrating cells attached to the lower side were fixed in methanol for 10 minutes and stained with 0.05% crystal violet for 20 minutes. The cells were observed under a microscope and cell numbers in 3 random fields in the middle of the membrane were counted.

### Statistical methods

Statistical analysis was performed using SPSS 20.0 statistical software. All data were tested for normality. Quantitative data with normal distribution was represented as mean ± SD of triplicate samples and differences between groups were tested using t-test and the analysis of variance (ANOVA). The Mann-Whitney U test was used for the comparisons of skew distributed data. The Chi-square test was used for the comparisons of categorical data between groups. Correlation between variables was analyzed using Pearson correlation analysis. Kaplan-Meier analysis and Log-rank test were used to assess differences in patient survival. All statistical tests were two-sided tests, and p<0.05 was considered as statistically significant.

## Results

### HIF-1α promotes the metastasis of esophageal cancer

**Figure 1:**
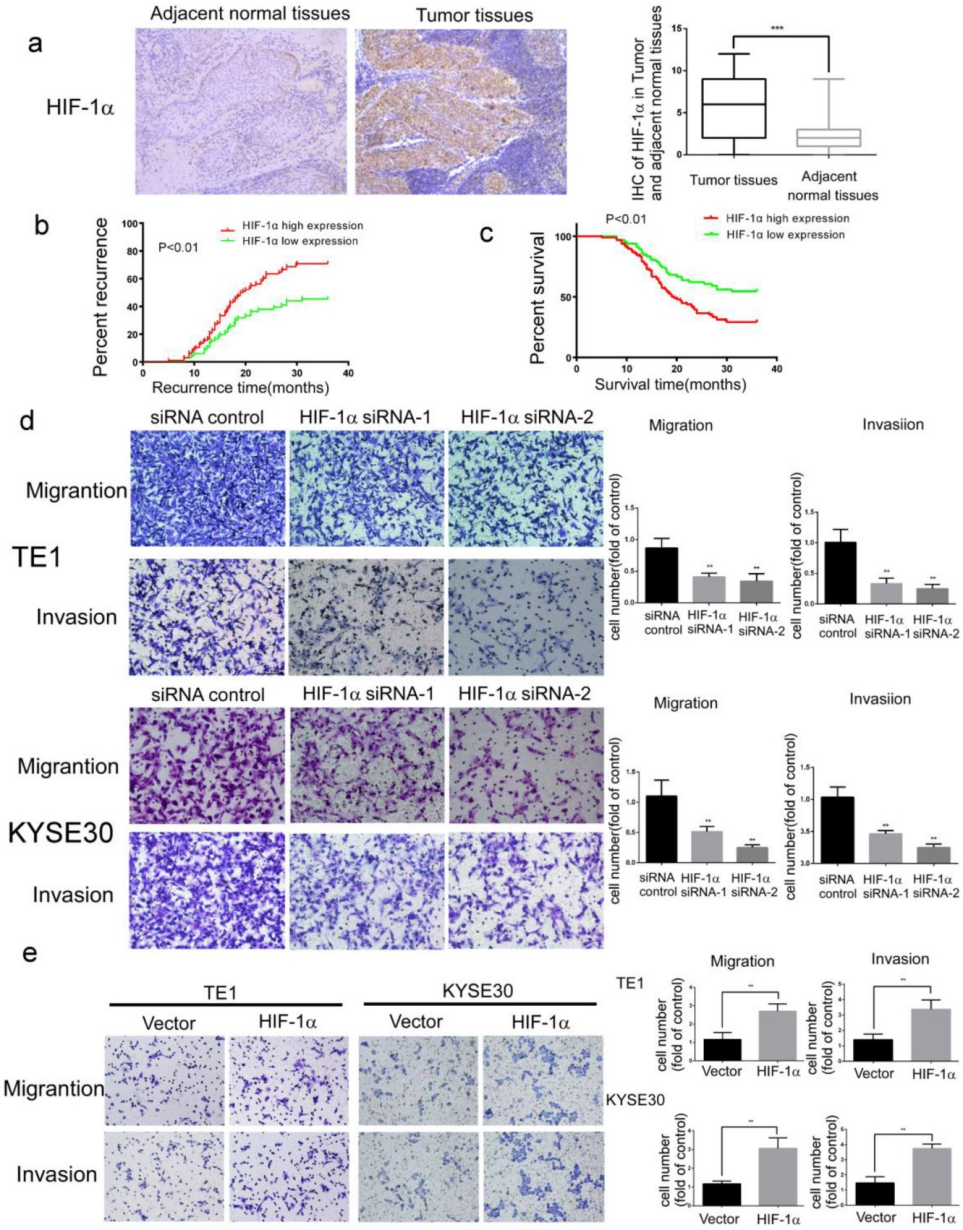
** The expression of HIF-1α in ESCC tissues and the functions of HIF-1α in ESCC cells. a** The expression of HIF-1α in ESCC detected by IHC, and the immunoreactivity score of HIF-1α in ESCC tumor tissues and adjacent normal tissues(n=182); **b** the 3-year regional recurrence curve of patients with HIF-1α high/low expression; **c** the 5-year survival curve of patients with HIF-1α high/low expression; **d** silencing *HIF-1α* by siRNA significantly suppressed the migration and invasion of TE1 and KYSE30, (n=3). Data represent the mean ± S.D; **e** overexpression of *HIF-1α* promoted migration and invasion of TE1 and KYSE30, (n=3). Data represent the mean ± S.D. (** P<0.01, *** P<0.001).

For the study of HIF-1α functions in esophageal cancer development, samples of cancer tissues and adjacent normal tissues were collected from 182 patients with ESCC. The results of immunohistochemistry assays showed that the expression of HIF-1α protein was significantly higher in ESCC than in adjacent normal tissues (Figure 1a and Table 1). In addition, the expression of HIF-1α in cancer tissues was associated with metastasis and recurrence (Table 2). Kaplan-Meier analysis showed that compared to those with lower HIF-1α expression, the patients with higher HIF-1α expression have higher rates of early metastasis and recurrence (estimated 3-year recurrence rate70.83% vs 45.46%, Figure 1b) and lower survival (estimated 5-years survival rate 8.15% vs20.25%, Figure 1c).

The migration and invasion ability of tumor cells is critical for tumor metastasis. In further analysis of the effect of HIF-1α on the migration and invasion of ESCC cells, it was found that the migration and invasion of ESCC cells were significantly inhibited by siRNA silencing of HIF-1α (Figure 1d). In contrast, significantly enhanced cell migration and invasion were observed upon overexpression of HIF-1α (Figure 1e).

These results indicated that HIF-1α might play an important role in metastasis, recurrence and poor prognosis of ESCC patients.

### HIF-1α promoted migration and invasion of ESCC cells by targeting Sp1

The above results confirmed that HIF-1α is an essential factor for the development of ESCC. It has been reported that HIF-1α regulates the expression of various target genes as transcription factor, therefore it is likely that its function in promoting metastasis of esophageal cancer is also working through the activation of downstream target gene transcription.

**Figure 2:**
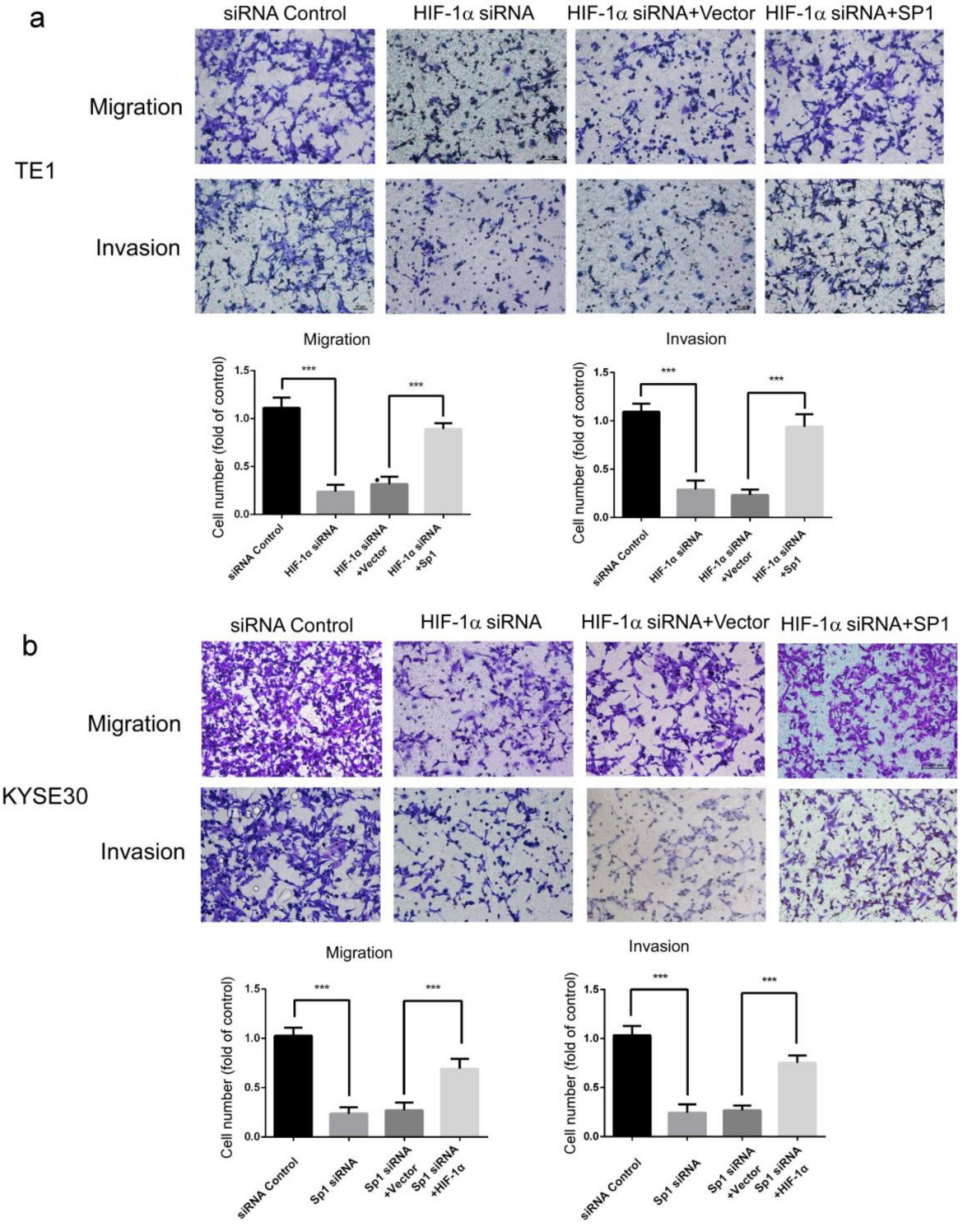
** Effects of SP1 overexpression on the migration and invasion of hypoxic ESCC cells. a, b** migration and invasion of TE1 and KYSE30 cells can be blocked by HIF-1α silencing, and can be rescued by SP1 overexpression (n=3). Data represent the mean ± S.D. (*** P<0.001).

Potential downstream target genes of HIF-1α were predicted in the UCSC and JASPAR databases and predicted candidate genes were further screened for those closely related to metastasis of ESCC. Among these genes, *SP1* has been reported to be involved in multiple mechanisms related to metastasis of ESCC 25-28. So we suspect that HIF-1α may regulate migration and invasion through SP1. The role of SP1 in HIF-1α-regulated migration and invasion of esophageal cancer cells were then investigated using complementation assays. The results showed that overexpression of SP1 significantly restored the migration and invasion of ESCC cells which was inhibited by HIF-1α silencing (Figure 2ab). These results indicated that HIF-1α regulated the migration and invasion of esophageal cancer cells by targeting SP1, and SP1 may be a critical factor in the regulation mechanism of HIF-1α.

### HIF-1α regulates SP1 expression through directly binding to the *SP1* promoter

**Figure 3:**
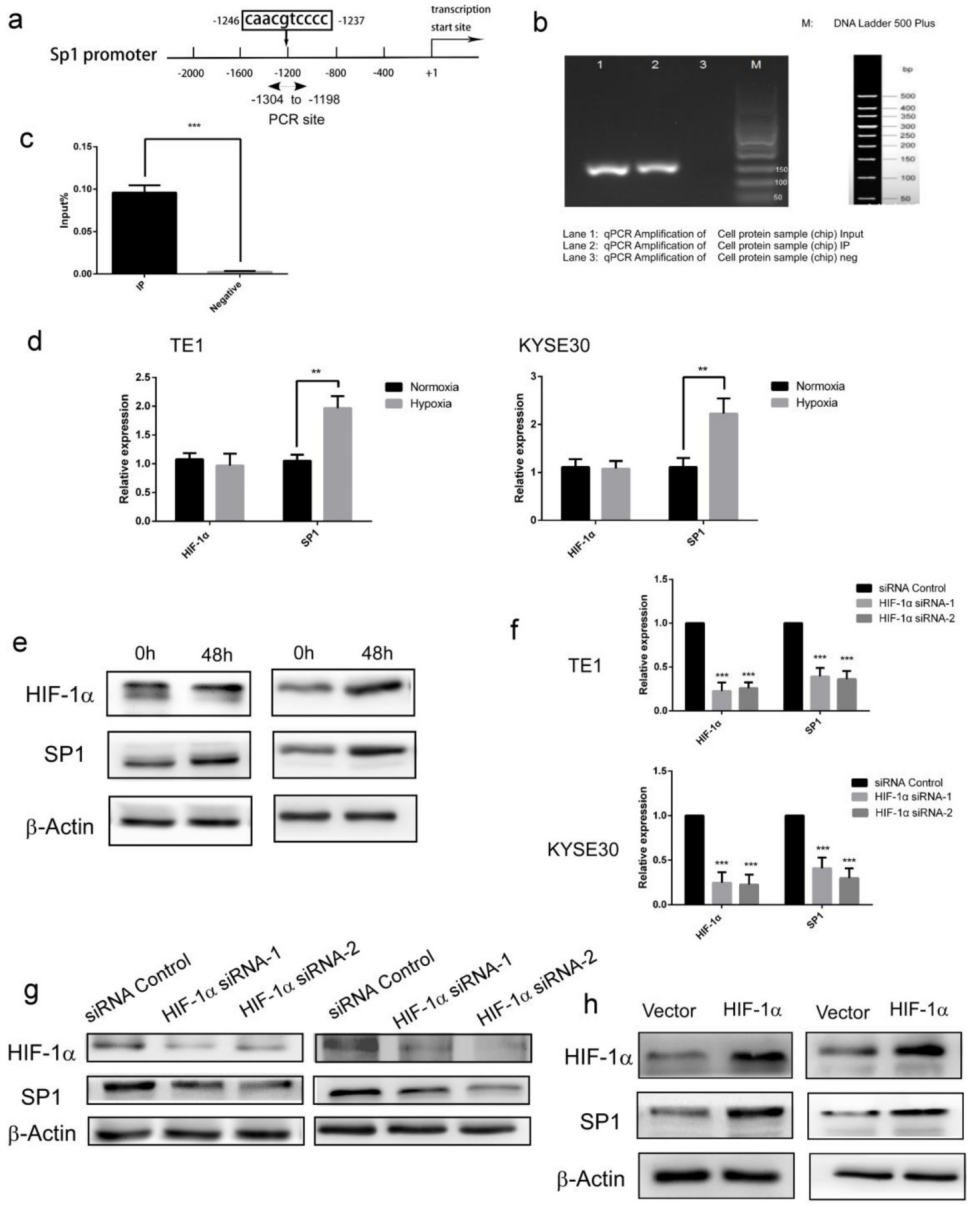
** HIF-1α up-regulates the protein expression of SP1 and its transcriptional activity by directly binding to the SP1 promoter. a** Schematic diagram depicting the positions of the primers used for the ChIP assay; **b, c** ChIP analysis was performed using a negative control immunoglobulin G (IgG) or anti- HIF-1α antibody in TE1 cells. Input positive control (anti- ARRDC3 antibodies); **d** HIF-1α and SP1 mRNA levels under both normoxic and hypoxic conditions were analyzed by real-time PCR. β-Actin was used as the internal control; **e** HIF-1α and SP1 protein levels under both normoxic and hypoxic conditions(48h) were analyzed by Western blotting. β-Actin was used as the internal control; **f** a knockdown efficiency of *HIF-1α* siRNA, and the mRNA levels of SP1 were detected by real-time PCR. β-Actin was used as the internal control (n=3);** g** a knockdown efficiency of *HIF-1α* siRNA, and the protein levels of SP1 were analyzed by Western blotting. β-Actin was used as the internal control (n=3); **h** the efficiency of over expression of *HIF-1α,* and the protein levels of SP1 were analyzed by Western blotting, the mRNA levels of SP1 were detected by real-time PCR. β-Actin was used as the internal control (n=3). (** P<0.01, *** P<0.001).

Next, we further studied the molecular mechanism of HIF-1α regulating SP1 expression. Forecast by UCSC and JASPAR database, A possible binding site (Figure 3a) of HIF-1α was identified in the *SP1* promoter (-1246 to -1237 bp). In subsequent Chip-PCR assay performed with extracts from ESCC cell lines, significant enrichment of *SP1* promoter sequence was achieved through immunoprecipitation with anti-HIF-1α antibody, but not with control IgG (Figure 3bc). These results indicated that as a transcription factor, HIF-1α could directly bind to the promoter of *SP1*. The regulatory effect of HIF-1α on SP1 was then verified in the esophageal cancer cell TE-1. The expression of HIF-1α and SP1 was firstly monitored under hypoxic conditions (48h). The results of qRT-PCR and Western blot showed that there was no significant change in the transcripts of *HIF-1α* in hypoxic condition, but the protein level was enhanced, this result is consistent with the literature 29. While, both the transcription and protein level of SP1 were increased significantly (Figure 3de). These data indicated that hypoxia enhances protein expression of HIF-1α and SP1 in ESCC cells. Upon siRNA silencing of HIF-1α, the mRNA levels of HIF-1α and SP1 were significantly decreased (detected with qRT-PCR, Figure 3f), as well as the protein levels (detected with Western blot, Figure 3g). Upon overexpression of HIF-1α, the mRNA and protein levels of *SP1* were significantly increased (Figure 3h). These data indicated that HIF-1α could directly regulate the transcriptional level of *SP1,* therefore affect its protein expression level.

### Expressions of HIF-1α and SP1 are positively correlated, and associated with metastasis, recurrence as well as poor prognosis

**Figure 4:**
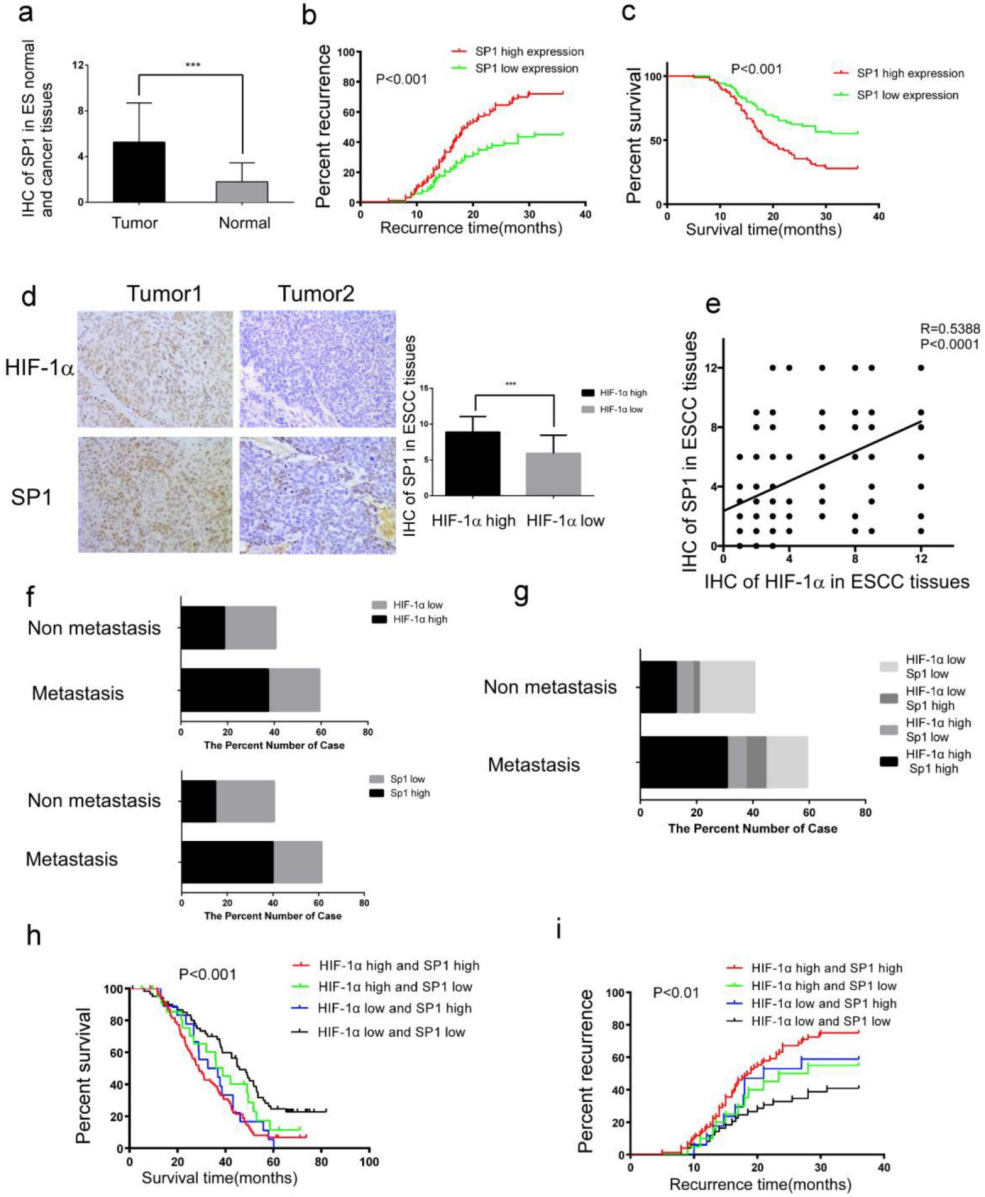
** Co-expression of HIF-1α and SP1 in ESCC tissues. a** The expression of SP1 in ESCC tissues detected by IHC, the immunoreactivity score of SP1 in ESCC tumor tissue and adjacent normal tissue (n=182); **b** the 3-year regional recurrence curve of patients with SP1 high/low expression; **c** the 5-year survival curve of patients with SP1 high/low expression; **d** the expression of HIF-1α and SP1 in ESCC detected by IHC, the immunoreactivity score of SP1 in HIF-1α high expression(n=102) and HIF-1α low expression(n=80) groups; **e** the correlation between the expression of HIF-1α and SP1 in esophageal cancer tissues; **f, g** the percent number of high/low expression of HIF-1α and SP1 in metastatic and non-metastatic groups; **h** the 5-year survival curve of patients with HIF-1α and SP1 positive/negative expression; **i** the 3-year regional recurrence curve of patients with HIF-1α and SP1 positive/negative expression. (** P<0.01, *** P<0.001).

To elucidate the relation between HIF-1α and SP1 in ESCC tissues, we examined the expression of SP1 in 182 ESCC tissues and adjacent normal tissues by immunohistochemistry. It was found that the expression of SP1 was higher in the ESCC tissues than in adjacent normal tissues (Figure 4a, Table 3). As shown in Table 2, high expression level of SP1 was significantly associated with tumor metastasis and recurrence (Table 2). Kaplan-Meier analysis estimated that the 3-year recurrence rate in patients with positive and negative expression of SP1 was 72.04% and 44.93%, respectively (Figure 4b), and the 5-year survival rate in patients with positive and negative expression of SP1 was estimated as 6.385% and 22.382%, respectively (Figure 4c). The immunohistochemical score of SP1 (Figure 4d) was significantly higher in HIF-1α high expression group (8.887±2.170) than in HIF-1α low expression group (5.913±2.531). There was a positive correlation between the expression of HIF-1α and SP1 in esophageal cancer tissues (R=0.5388 P <0.0001, Figure 4e).

Among 108 ESCC patients with tumor metastasis, 68 patients (62.9%) had high expression of HIF-1α, 69 patients (63.9%) had high expression of SP1; while among 74 patients without tumor metastasis, 34 patients (45.9%) were with high expression of HIF-1α and 27 patients (36.5%) had high expression of SP1 (Figure 4f). Both HIF-1α and SP1 were positively associated with tumor metastasis (P<0.001, Table 2). Among the 108 patients with tumor metastasis, 56 patients (51.8%) had high expression of both HIF-1α and SP1, 12 patients (11.1%) had high expression of HIF-1α, 13 patients (12.0%) had high expression of SP1, and 27 patients (25.0%) patients had low expression of both HIF-1α and SP1 (Figure 4g). Patients with high expression of both HIF-1α and SP1 were more likely to have tumor metastasis (Table 4).

Kaplan-Meier analysis estimated that patients with high expression of both HIF-1α and SP1 had a higher 3-year recurrence rate of 75.0% (Figure 4h), and lower 5-year survival rate of 12.11% (Figure 4i), compared with the others.

The above results indicated that there was a positive correlation between HIF-1α and SP1 levels in esophageal cancer tissues, and concurrent expressions of these two proteins were associated with poor prognosis.

## Discussion

ESCC is a highly invasive tumor. Metastasis is the major-risk factor affecting the prognosis of ESCC patients, especially Lymph node metastasis, with metastasis as the most common cause of surgical treatment failure. Therefore, research in tumor metastasis mechanism of esophageal cancer is of great significance for the exploration of novel target to improve treatment effect and prognosis of esophageal cancer patients. However, the related mechanism about metastasis of esophageal cancer is still unclear.

Studies till present indicate that the main cause of metastasis of ESCC is the generation of blood vessels and lymphatics in the tumor microenvironment, which in turn promotes the metastasis of tumor cells to lymph nodes and distant organs 30-32. HIF-1α is activated in hypoxic environment, and regulates transcription of downstream genes involved in various cell functions, including proliferation, survival, cell migration, glucose metabolism, pH regulation, angiogenesis and lymphangiogenesis, allowing cells to adapt to hypoxic environment, contributing to proliferation and highly invasive and metastatic ability 33-36. The hypoxic tumor microenvironment induces increase in the transcriptional activity of HIF-1α, which in turn stimulates transcription of some genes associated with angiogenesis and lymphangiogenesis 30, 31. Although it has been observed that the density of blood vessels and lymphatics is associated with high expression of HIF-1α in different malignant tumors, the prognostic value of these features in tumors from squamous epithelium is still controversial 37. This study validated the relationship between HIF-1α expression and tumor metastasis in ESCC and the effect of HIF-1α on the prognosis of patients with ESCC.

Studies have shown that HIF-1α is associated with tumor metastasis in thyroid papillary carcinoma 38. T.Kurokawa et al 7 showed that high expression of HIF-1α was positively correlated with tumor metastasis and poor prognosis in patients with ESCC. This was consistent with our results and indicated that HIF-1α plays an important role in metastasis of ESCC, although the molecular mechanism remains to be further clarified. In this study, we found that patients with high expression of both HIF-1α and SP1 in ESCC tissues were with higher recurrence rate and lower survival rate, this suggests that HIF-1α plays an important role in the metastasis and recurrence of ESCC with SP1.

Previous studies have confirmed that SP1 is highly expressed in gastric cancer, pancreatic cancer, etc. 39-41, related to high level of tumor malignancy and poor prognosis, which could accelerate tumor metastasis, promote epithelial-mesenchymal transition and regulate cell cycle 42, 43. However, there still remains controversy about the functions of SP1, some reports expound that SP1 plays an important role in anticancer 44-46. So, it is still unclear whether SP1 plays a dual role in the development of cancer, which requires further investigation. We just suspect that SP1 may change its transcriptional activity at different tumor stages. In this study, we found that SP1 is highly expressed in ESCC, and accelerates tumor metastasis.

At present, there are few studies on the mechanism of SP1 in metastasis of ESCC. Studies on the potential interaction mechanism between HIF-1α and SP1 have been reported in human glioblastoma 47 and CNS ischemia in rat 48. However, it is unclear whether there is a specific interaction between HIF-1α and *SP1* promoter in tumor cells.

In this study, we analyzed the UCSC and JASPAR database and found that HIF-1α may bind to the *SP1* promoter region (-1246 to -1237 bp) and verified the binding using ChIP assay. It was also proved that HIF-1α could directly regulate the expression of SP1 by binding to the promoter of *SP1*, thereby affecting the metastasis of ESCC. The concurrent high expressions of HIF-1α and SP1 were found to be associated with metastasis of ESCC (P<0.01 Table 4).

Activation of VEGF-C and VEGF-A in tumor microenvironment is critical for lymphangiogenesis and angiogenesis 48, 50. Studies have shown that HIF-1α could promoted the expression of VEGF-A 51, and it has been reported that SP1 could promote the expression of VEGF-C in breast cancer 52. These results suggested that HIF-1α/SP1/VEGF regulatory pathway might be an important molecular mechanism in the metastasis of tumors.

In summary, this study has found that HIF-1α and SP1 are highly expressed in esophageal cancer tissues and the expression levels of these two are positively correlated. Concurrent high expression of these two proteins is closely related to tumor metastasis and poor prognosis. It is also confirmed that HIF-1α has a direct regulatory effect on the transcription of SP1 in esophageal cancer. Our results may help elucidate the molecular mechanism of tumor metastasis and recurrence in ESCC and provide a new theoretical basis for targeted therapy.

## Figures and Tables

**Table 1:** The expression of HIF-1α in ESCC and adjacent normal tissues

ESCC	Case n	Expression of HIF-1α		*X^2^ value*	*P* value
		Low	High		
**Tumor tissues**	182	80	102	87.72	<0.001
**Adjacent normal tissues**	182	164	18		

**Table 2:** Clinical characteristics and its relationship with HIF-α/ Sp1 expression

Clinical characteristic	HIF-1α high expression	p value	Sp1 high expression	p value
**Gender**		0.82		0.10
Male	91/161		81/161	
Female	11/21		15/21	
**Age**		0.18		0.66
≥60	44/87		44/87	
<60	58/95		52/95	
**Invasion depth**		0.40		0.06
T1	5/13		3/13	
T2	33/59		30/59	
T3+T4a	64/110		63/110	
**Lymphatic metastasis**		0.023		<0.001
yes	68/108		69/108	
no	34/74		27/74	
**Differentiation**		0.12		0.88
well	37/55		28/55	
moderate	31/63		32/63	
low	34/64		30/64	
**Recurrence**		0.045		0.001
yes	55/88		59/88	
no	27/59		24/59	

**Table 3:** The expression of SP1 in ESCC and adjacent normal tissues

ESCC	Case n	Expression of Sp1		*X^2^ value*	*P* value
		Low	High		
Tumor tissues	182	86	96	95.64	<0.001
Adjacent normal tissues	182	171	11		

**Table 4:** Clinical characteristics and its relationship with HIF-α & Sp1 expression

Clinical characteristic	HIF-1α high Sp1 high	HIF-1α high Sp1 low	HIF-1α low Sp1 high	HIF-1α low Sp1 low	p value
**Gender**					0.06
Male	69/161	22/161	12/161	58/161	
Female	10/21	1/21	5/21	5/21	
**Age**					0.36
≥60	36/87	8/87	8/87	35/87	
<60	43/95	15/95	9/95	28/95	
**Invasion depth**					0.22
T1	2/13	3/13	1/13	7/13	
T2	27/59	6/59	3/59	23/59	
T3+T4a	50/110	14/110	13/110	33/110	
**Lymphatic metastasis**					0.003
yes	56/108	12/108	13/108	27/108	
no	23/74	11/74	4/74	36/74	
**Differentiation**					0.21
well	28/55	9/55	4/55	14/55	
moderate	26/63	5/63	4/63	28/63	
low	25/64	9/64	9/64	21/64	
**Recurrence**					0.019
yes	40/88	15/88	12/88	21/88	
no	22/59	5/59	4/59	28/59	
